# A comparison between the preferences for oral corrective feedback of teachers and students of Chinese as a second language

**DOI:** 10.3389/fpsyg.2023.1112136

**Published:** 2023-03-09

**Authors:** Rui Bao, Hui Wang

**Affiliations:** College of International Culture and Social Development, Zhejiang Normal University, Jinhua, China

**Keywords:** oral corrective feedback, teacher and student preference, Chinese as a second language, error types, the efficacy of feedback

## Abstract

This study reports on the preferences of teachers and students of Chinese as a second language (CSL) for corrective feedback (*CF*) and the reasons underlying their preferences. Data was collected from 328 students and 46 teachers through a questionnaire and interviews, and the results showed that CSL students had a strong overall preference for explicit correction and metalinguistic clues, while teachers favored recasts. Moreover, students and teachers significantly varied in their preferences for metalinguistic clues, explicit correction, and clarification requests across error types. A difference was also identified in recasts regarding phonological and lexical errors. Explanations for these variations are attributed to the characteristics of the Chinese language, learner proficiency, teachers’ entrenched beliefs in teaching, and the features of some given *CF* types. Additionally, the interview data also revealed the different reasons that teachers and students consider when it comes to *CF* provision. Finally, pedagogical implications for language teachers are discussed.

## 1. Introduction

Corrective feedback (hereafter *CF*) refers to “responses to learner utterances containing an error” (Ellis, 2006, p. 28) in both oral and written modes. The focus of the present study is on oral *CF*; over the past two decades oral *CF* has been the subject of considerable research in the field of second language education. Earlier scholars focused on the critical role of oral *CF* in preventing learners from fossilizing their L2 errors, a phenomenon considered damaging to L2 learning (DeKeyser, 1993), while subsequent scholars have highlighted the role of *CF* in stimulating learners to notice gaps between their interlanguage and the target norm, prompting them to reformulate their utterances in a more target-like manner (Long, 1996). Increasing evidence has revealed the overall efficacy of *CF* on L2 learning (Li, 2010; Lyster et al., 2013), but it has been also found that the pattern and efficacy of *CF* relate to various contextual variables (Zhu and Wang, 2019). In recent years, teacher and student beliefs as one variable has attracted much attention in *CF* research (Ha and Murray, 2023). Beliefs refer to the attitudes, views, opinions, or stances that teachers and learners hold about the tasks or strategies used in second language teaching and learning (Li, 2017). Given the difficulty in distinguishing beliefs from other constructs such as perceptions, attitudes, preferences, etc., the term belief in the present study is used interchangeably with these constructs.

It has been long recognized that teacher and student beliefs about L2 teaching and learning exert a profound impact on their perceptions and judgments, which in turn influence what they do and say during classes (Borg, 2003). In addition, the relationship between the two belief systems also plays a big role in L2 learning, with consistency leading to “harder work and greater gains in students’ learning” (Plonsky and Mills, 2006, p. 55), while inconsistency may result in students’ dissatisfaction, anxiety, and even the cessation of further study (Horwitz, 1999; Brown, 2009). Importantly, belief as a social construct is context-dependent (Barcelos, 2003), which means that teachers and students from different contexts may vary in their beliefs about L2 teaching and learning. Thus, it merits more inquiries, which is particularly true in the context of Chinese as a second language (CSL), since knowledge about this area is scant.

The present study seeks to examine CSL teachers’ and students’ preferences for different *CF* types regarding grammatical, phonological, and lexical errors and the reasons for their *CF* preferences. Data was collected by means of questionnaires and interviews. The results are expected to yield an in-depth understanding of CSL teachers’ and students’ *CF* preferences so as to inform teachers about how to make their *CF* practices more effective and efficient for teaching and learning.

## 2. Literature review

### 2.1. Research on oral corrective feedback

One seminal study on oral *CF* was carried out by Lyster and Ranta (1997), identifying six types of oral *CF*: recasts, explicit correction, metalinguistic clues, repetition, elicitation, and clarification requests. According to whether the correct form was directly provided or withheld, they further categorized these types of *CF* into two broad groups: reformulations (i.e., recasts and explicit correction), and negotiation of forms (i.e., elicitation, metalinguistic clues, clarification requests, and repetition), also referred to as prompts (Lyster, 2004). Another way to segment these *CF* types was given by Ellis et al. (2006), who classified them into explicit and implicit types according to whether the learner was drawn to their error overtly. In this segmentation, implicit *CF* constitutes clarification requests, repetition, recasts, and elicitation, while explicit *CF* includes metalinguistic clues and explicit correction.

Despite the existence of different classifications, Lyster and Ranta’s taxonomy has been widely used to code *CF*. To date, extant *CF* research falls mainly into two paradigms: observational and experimental (Li and Vuono, 2019). Research in the observational line has focused on describing *CF* patterns in terms of the distribution of different types of *CF* and their links to learner uptake (Brown, 2016; Wang and Li, 2020), while experimental research has emphasized *CF* efficacy in regards to whether *CF* is conducive to L2 learning and which type is most effective (Li, 2010; Lyster et al., 2013; Nassaji, 2017). In summary, both lines of research have shown a clear tendency for the efficacy of *CF* on L2 learning and also noted a host of mediating factors contributing to the patterns and efficacy of *CF* (Nassaji and Kartchava, 2020), of which is teacher and student preferences for *CF*.

### 2.2. Teacher and student preference for corrective feedback

Early research on teachers’ and students’ preferences for *CF* was mainly conducted with broader surveys of general beliefs about L2 teaching and learning, using Likert-type questionnaires that included only a few items related to *CF* (Schulz, 2001; Davis, 2003; Brown, 2009). However, more recent research has focused exclusively on *CF* beliefs in terms of attitudes, perceptions and preferences, the results of which have shown that teachers’ and students’ beliefs do not always align (Lee, 2013; Kaivanpanah et al., 2015; Roothooft and Breeze, 2016). For instance, Ha and Nguyen (2021) found that students are disposed toward having *CF* for all error types and desired to use peer correction during class. However, teachers were found to have less confidence in peer correction and more reluctance in giving *CF*. There are also discrepancies between teachers’ and students’ beliefs concerning *CF* timing, as Ha et al. (2021) reported that Vietnamese students appreciated immediate *CF* while their teachers were less favorable about this *CF* techniques out of concern for students’ emotional state and disruption of *CF* on the flow of students’ speech.

In addition, discrepancies have also been identified between teachers’ and students’ preferences for different types of *CF*. For instance, Yoshida (2008) showed that Japanese EFL learners preferred to be given time to rethink and self-repair their errors, as opposed to their teachers’ preference for recasts. ESL teachers in Lee (2013) also favored recasts, but their advanced-level learners preferred explicit correction. This also held true for Roothooft and Breeze (2016), who revealed that Spanish learners of EFL appreciated explicit *CF* in terms of explicit correction and metalinguistic clues, but their teachers preferred elicitation. However, unlike the studies reviewed above, Kaivanpanah et al. (2015) found that Iranian EFL learners showed a positive view of recasts and metalinguistic clues, while their teachers tended to use different types of *CF* depending on the circumstances. Taken together, this research shows that teachers and students in different contexts may hold varied preferences for *CF* types.

Explanations for these differing preferences have been related to a number of contextual variables, proficiency being one of them. Brown (2009) revealed high-proficiency students were more favorable to being corrected indirectly than lower-proficiency students. Similar findings were also found by Kaivanpanah et al. (2015), who noted that Iranian learners with higher proficiency responded more positively to elicitative types of *CF* that required self-correction compared to those with lower proficiency. Another study worth noting is Zhang et al. (2022), which compared *CF* beliefs between high- and low- proficiency CSL learners and found that low-proficiency learners preferred delayed correction more than those with high proficiency. Furthermore, proficiency also affects learners’ preferences for *CF* regarding error types, as Yang (2016) indicated that intermediate CSL learners are more confident in the efficacy of clarification requests on pronunciation errors than beginning learners. In addition, individual learning experiences (Schulz, 2001; Loewen et al., 2009), instructional settings (Lee, 2013; Kaivanpanah et al., 2015), and learner affect (Yoshida, 2008) also have contributed to the variance of *CF*-related preferences. This again highlighted the context-specific nature of beliefs, which suggests a need for more inquiries to examine the effects of other factors on teacher and learner *CF* preferences.

### 2.3. Effect of error types on teacher and student preference for corrective feedback

Previous research has shown that error type is a factor affecting learners’ perceptions of *CF*. For instance, a number of studies have reported a tendency for learners to perceive phonological and lexical *CF* more accurately than grammatical *CF* (Nabei and Swain, 2002; Yoshida, 2010). However, there are exceptions, as Mackey et al. (2007) reported that learners of Arabic were more likely to notice teachers’ *CF* of lexical and grammatical errors than phonological ones. In addition, there is also a discrepancy between learners’ perceptions of and teachers’ intentions for *CF* with respect to error types. This line of research has shown an overall tendency that teachers tend to provide more *CF* for grammatical errors, while learners perceive phonological and lexical errors more accurately (Mackey et al., 2000). Discrepancy is also noted between teachers’ preferences for and learners’ responses to *CF*. This was demonstrated by Lyster (1998) who discovered that teachers preferred to recast grammatical errors, whereas learners generated more successful grammatical repairs as a result of negotiation of forms. Nonetheless, *CF* research in the context of Chinese as a second language is rather limited, especially in terms of comparisons between teachers’ and learners’ preferences for different types of *CF* and the underlying reasons. This dearth of material makes the present study essential.

### 2.4. Research on Chinese as a second language

Mainstream CSL research has long focused on the characteristics of the Chinese language from a linguistic perspective. Over the past decade, with the growing number of Chinese language learners and the global demand for effective Chinese pedagogy both in and outside China (Orton, 2008; Moloney and Xu, 2015), increasing attention has been paid to issues related to classroom teaching and learning, *CF* being one of the most prominent. It has been found that as with other languages, recasts are also the most frequently used type of *CF* in CSL classrooms (Bao, 2019), and the efficacy of *CF* on CSL learning has also been confirmed (Lu and Gao, 2015). Regarding the efficacy of different types of *CF*, prompts have been found to be more effective than recasts in the long term (Cao and Mu, 2013), echoing the findings of Ammar and Spada (2006). Likewise, variance also exists, as some studies show that phonological errors received *CF* most (Bao, 2019), whereas other research reports that CSL teachers provide the largest amount of *CF* for grammatical and lexical errors (Duan and Sun, 2015). In addition, Yang (2016) explored CSL learners’ preferences for six types of *CF* in relation to specific error types and found learners’ general preferences for explicit correction, metalinguistic clues, and recasts across error types. This research casts some light on *CF*-related beliefs in the context of *CF*. However, research on comparing CSL teachers’ and learners’ preferences for *CF* is scant. This justifies the need for the present study, especially considering the features of the Chinese language and the source of Chinese teachers’ pedagogical beliefs.

It is widely accepted that the unique sound, grammar, and orthography systems of the Chinese language not only distinguish it from many other languages but present challenges for foreign learners, in particular its pronunciation and tone (Orton, 2008). These linguistic features might lead to different preferences for *CF* between CSL students and those of alphabetic languages. Additionally, it is well-known that Chinese teaching beliefs have been significantly influenced by Confucian thoughts (Hu, 2002). As a result of this influence, the teacher is seen as an authority figure, taking on primary responsibility to transmit knowledge and supervise learner performance, while students are expected to be obedient and maintain a high level of receptiveness (Li, 2003). This conceptualization of the teacher’s role may lead to Chinese teachers’ *CF* preferences differing from their counterparts under the Western education schema and students coming from a background unrelated to Confucian culture. The reasons outlined above provide another justification for the need for the present study by focusing on the following research questions:

(1) What are CSL teachers’ and students’ preferences for *CF* types in terms of grammatical, lexical, and phonological errors?
(2) Are there any significant differences between CSL teachers’ and students’ preferences for *CF* types?
(3) What might account for the CSL teachers’ and students’ respective preferences for *CF*?

## 3. Methodology

### 3.1. Instructional context

This study was conducted at two southern Chinese universities, which were selected through convenience sampling, since the authors worked in one, and had a personal connection with the other. Both universities provided a Chinese language program with an aim to help foreign students’ improve their Chinese proficiency. The respective programs at both universities had the same curriculums, textbooks, teaching methods, and assessment techniques. Specifically, they included listening, speaking, reading, writing, and comprehensive Chinese courses. In order to accommodate individual participant proficiency, three levels of modules were established according to students’ placement tests; these were elementary, intermediate, and advanced, each of which had two subcategories (i.e., lower and upper). All modules constituted 4 to 6 teaching sessions per day. Teaching at both universities was delivered by full-time university teachers, part-time teachers, and second-year intern graduate students majoring in Teaching Chinese to Speakers of Other Languages. A series of textbooks titled ‘*Developing Chinese*’ was used for all courses. At the end of each semester there was a written test, which was designed by the corresponding teacher of each course.

### 3.2. Participants

A total of 328 students and 46 teachers participated in this study. The students came from various cultures; over half of them (53.8%) were from Asia, with African students being the next largest group, and a small percent from Western nations. Among them, there were 193 females and 135 males. They aged from 18 to 39 years old, with a mean age of 34 years. In terms of Chinese proficiency, students varied in their HSK level^1^, ranging from HSK1 to HSK6. Specifically, participants with HSK1-3 were classified as elementary (*n* = 120), those with HSK4–6 were treated as intermediate (*n* = 208). Despite these differences, all the participants at both universities studied in a similar language program. Meanwhile, they shared a target-language learning context, which exposed them to plentiful Chinese input and had access to use Chinese in their daily life. In addition, as reported in the interviews, participating students have not been treated differently in class regardless of their various backgrounds. Given these commonalities, participating students were treated as one group. Certainly, any differences will be reported.

As for the 46 teachers, 34 of them were female and the rest (*n* = 12) were male. All were full-time, and their age ranges from 25 to 60 years old with a mean age of 40 years. They all had more than 5 years of experiences in teaching CSL, classifying them as experienced teachers according to Gatbonton (2008). All majored in Chinese linguistics; some of these (*n* = 15) were at PhD level while the rest (*n* = 31) were Masters. As such, the teachers were also treated as one group.

### 3.3. Data collection

In order to capture the varying and dynamic nature of beliefs, the present study drew on the methods of questionnaires and interviews.

#### 3.3.1. Questionnaires

A Likert-type questionnaire was used for this study given the efficacy of this technique in eliciting respondent beliefs on a large scale (Barcelos, 2003). Both teachers’ and students’ questionnaires had two parts. Part one in students’ and teachers’ questionnaires each had six questions related to their backgrounds. Part two in both questionnaires was mainly adapted from Yang (2016), which constituted 18 Likert-type questions focusing on teachers’ and students’ preferences. In order to make comparison in the responses between teachers and students, these questions in both questionnaires were presented identically and in the same order. Respondents were asked to rate each question on a 4-point Likert-type scale, with 1 being the most negative and 4 being the most positive. The teachers’ questionnaire was written in Chinese, as all of them are native Chinese speakers. As for the students’ questionnaire, Part one was presented in English, while Part two was in both Chinese and English together in order for students to understand the specific examples of errors and the given *CF*. To ensure the validity of the two questionnaires, the initial versions were pilot-tested with 55 students and 15 teachers, respectively. Comments from these pilot samples were incorporated and three questions were reworded for clarity. The final questionnaires (see Appendix A) were electronically administered to 420 students and 57 teachers, and the responses were received back within one week. After excluding some incomplete questionnaires, 328 questionnaire responses from students and 46 from teachers were used for this study. Cronbach’s alpha for teacher and student questionnaires were 0.781 and 0.873 respectively, indicating sufficient reliability.

#### 3.3.2. Semi-structured interview

Because of the drawbacks of Likert-type questionnaires in eliciting respondents’ deeper insights into the phenomenon under study (Barcelos, 2003), semi-structured interviews were also used given their utility in assessing people’s perceptions and opinions (Punch, 2009). Considering the difficulty of interviewing a large number of participants’ perspectives, 14 students, with different proficiencies and cultural backgrounds, and 8 teachers were randomly selected for the interview. Among these, 8 students and 4 teachers from the authors’ university took part in face-to-face interviews, and the rest from another university were interviewed online. The interviews were individual, which were all audio-recorded. The interview questions (see Appendix B) focusing on the reasons behind teachers’ and students’ *CF* preferences were presented naturally during the conversation between the interviewees and the first author. Each interview lasted for 15 to 20 min, all of which was transcribed by the first author. Tables 1, 2 present the teacher and student interviewees’ backgrounds, respectively.

**Table 1:** The teacher interviewees’ backgrounds.

Name	Age	Gender	Major	Degree	Years of CSL teaching	Overseas teaching experiences
Sun	44	Female	Chinese linguistics	PhD	19	0
Li	43	Female	Chinese literature	PhD	6	0
Wang	47	Female	Chinese linguistics	MA	12	0
Ruan	51	Female	Chinese linguistics	MA	14	0
Qian	28	Female	Chinese linguistics	MA	5	4 years
Zhao	29	Male	Chinese linguistics	MA	5	4 years
Chen	45	Female	Chinese linguistics	MA	5	3 years
Hu	48	Female	Chinese linguistics	MA	12	6 years

**Table 2:** The student interviewees’ backgrounds.

No.	Age	Gender	Country	Years of CSL learning	Proficiency	Module
1	23	Male	Vietnam	4.5 years	HSK5	Advanced
2	24	Male	Madagascar	4 years	HSK5	Advanced
3	24	Male	Madagascar	4 years	HSK4	Intermediate
4	28	Male	America	3 years	HSK4	Intermediate
5	22	Female	Mexico	3 years	HSK4	Intermediate
6	25	Female	Vietnam	3.5 years	HSK4	Intermediate
7	22	Female	Thailand	2.5 years	HSK3	Elementary
8	23	Female	Thailand	2 years	HSK3	Elementary
9	25	Female	Sudan	3 years	HSK4	Intermediate
10	27	Female	Tanzania	4 years	HSK5	Advanced
11	22	Female	Hungary	2.5 years	HSK3	Elementary
12	24	Male	Spain	5 years	HSK6	Advanced
13	22	Female	Indonesia	3 years	HSK4	Intermediate
14	26	Male	Tanzania	5 years	HSK6	Advanced

### 3.4. Data analysis

In response to Question 1, SPSS22.0 was used to carry out descriptive statistics to calculate the mean scores of the 18 items asked in both questionnaires. To answer Question 2, an independent *t*-test was carried out to examine whether there are significant differences between the teachers’ and students’ *CF* preferences. As for Question 3, a qualitative analysis of the interview data was analyzed inductively and coding was grounded in the data. Firstly, we iteratively read through the students’ interview transcripts and used open coding to assign codes to pieces of data pertinent to the reasons for their *CF* preferences (Corbin and Strauss, 2008). These codes provided an index to store and retrieve the data by recursive examination. Secondly, through many rounds of repetitive reading of the transcripts and those assigned codes, we merged the related codes into categories and developed them into a number of themes. Once these themes were established, we pooled all data touching on these themes. Finally, we moved iteratively from the data to the established themes and conceptualized the ultimate themes for the current study through axial coding. The same coding process was also carried out on the teachers’ interview transcripts to identify the reasons underlying their *CF* preferences. To enhance the reliability of this coding, member checking was used by sharing the coding with each interviewee, who agreed with the interpretations of their responses.

## 4. Findings

### 4.1. Chinese as a second language teachers’ and students’ preferences for different types of corrective feedback

As for this question, the mean scores for the eighteen questions asked in the teacher and student questionnaires were calculated (see in Table 3). The results indicated CSL students’ preferences for explicit correction and metalinguistic clues across error types, mean scores of which were all over 3.00 (out of a maximum score of 4.00). However, they were less disposed toward repetition, elicitation, and clarification requests, with repetition having the lowest rating, regardless of error types. This seems to suggest CSL students’ preference for explicit types of *CF* over implicit ones. In terms of recasts, the mean scores for all three error types were relatively high, with phonological error being 2.79 and grammatical and lexical errors both being over 3.00. This seems to indicate that CSL students somewhat favored recasts, especially for the correction of grammatical errors (*M* = 3.27).

**Table 3:** Teachers’ and students’ preferences for different types of *CF*.

Category	Items	Grammatical error		Phonological error		Lexical error	
		Mean	SD	Mean	SD	Mean	SD
Learners’ beliefs	1. Explicit correction	3.21	0.841	3.40	0.696	3.26	0.780
	2. Recasts	3.27	0.730	2.79	0.913	3.02	0.844
	3. Metalinguistic clues	3.17	0.787	3.37	0.691	3.13	0.792
	4.Elicitation	2.62	0.953	2.63	0.987	2.67	0.942
	5. Repetition	2.59	0.967	2.51	0.929	2.52	0.945
	6. Clarification request	2.85	0.976	2.88	0.972	2.87	0.971
Teachers’ beliefs	1. Explicit correction	2.65	0.674	2.70	0.726	2.67	0.701
	2. Recasts	3.13	0.542	3.02	0.577	3.20	0.453
	3. Metalinguistic clues	2.54	0.657	2.83	0.677	2.48	0.722
	4. Elicitation	2.48	0.691	2.48	0.722	2.46	0.721
	5. Repetition	2.59	0.686	2.57	0.655	2.65	0.640
	6. Clarification request	2.41	0.858	2.39	0.774	2.48	0.722

With regard to CSL teachers, they showed strong preferences for recasts across error types, the mean scores all being over 3.00. However, they were less disposed toward explicit types of *CF* and prompts; the mean scores for all these types were around the baseline (*M* = 2.5).

### 4.2. Significant differences between the Chinese as a second language teachers’ and students’ preferences for corrective feedback types

To answer this question, an independent *t*-test was performed to examine whether there were significant differences between CSL teachers’ and students’ preferences for different types of *CF* regarding the three error types. The results identified 11 items that showed significant differences (see Table 4), which are only reported here briefly due to space limitations.

**Table 4:** Items with significant differences between the teachers’ and students’ preferences for *CF*.

Error type	*CF* types	Mean difference (Ss-Ts)	*t*	df	Sig. (2-tailed)
Grammatical error	4. Metalinguistic clue	0.63	5.159	372	0.000
	5. Explicit correction	0.56	4.335	372	0.000
	6. Clarification request	0.44	2.887	372	0.004
Phonological error	1. Recasts	−0.23	2.347	372	0.021
	3. Explicit correction	0.70	6.359	372	0.000
	5. Metalinguistic clue	0.54	4.974	372	0.000
	6. Clarification request	0.49	3.253	372	0.001
Lexical error	2. Recasts	−0.18	2.215	372	0.029
	3. Explicit correction	0.59	4.822	372	0.000
	4. Metalinguistic clue	0.65	5.287	372	0.000
	6. Clarification request	0.39	2.608	372	0.009

As can be seen, significant differences were found between CSL teachers and students with regard to their preferences for explicit correction, metalinguistic clues, and clarification requests across error types. Interestingly, as for these items, the mean scores from the CSL students were all higher than those from the teachers, indicating that CSL students have a greater preference for these types of *CF* than teachers, especially in terms of explicit correction and metalinguistic clues. Significant difference is also found in recasts with regard to phonological and lexical errors; in the case of both items, the CSL teachers were more confident than students. This indicates that CSL students approve more of explicit types of *CF* and clarification requests, while CSL teachers embrace recasts.

### 4.3. Reasons for the Chinese as a second language teachers’ and students’ corrective feedback preferences

To answer this question, a qualitative analysis of the interview transcripts from the teachers and students was conducted, concentrating on the reasons for their preferences for oral *CF*. The results were presented, respectively.

#### 4.3.1. Reasons for teachers’ corrective feedback preferences

In relation to the CSL teachers, the findings showed seven reasons behind their *CF* preferences. Table 5 gives a nuanced overview of these reasons, while illuminating examples of teachers’ comments for each reason are presented in Table 6.

**Table 5:** Reasons for the teachers’ *CF* preferences.

Coding categories	*of coded T Qu	*of coded instances in T Qu
Stressing learner participation in error correction process	6	18
Learner proficiency	4	7
Learner affect	8	19
Teachers’ individual experiences	2	2
The gravity of errors	6	9
Priority on the flow of students’ speech	4	5
Concern for instructional pace	1	1

*of coded T Qu refers to the number of questionnaires in which such a comment was coded; *of coded instances in T Qu refers to the number of comments that were coded, i.e., there could be several comments on a theme in one and the same questionnaire.

**Table 6:** Comments from the teachers for their *CF* preferences.

Coding categories	Examples
Stressing learner participation in error correction process Learner proficiency	“I think repetition is effective because it clearly directs to the error, thus raising learners’ awareness of their errors and further reflecting upon their errors.” (Sun) “I prefer clarification requests, as doing so can leave space for learners to rethink their utterances so as for them to remedy in a more target-like manner” (Wang) “For elementary learners, I do more correction, while I will invite other peer students to correct each other at intermediate or advanced level.” (Qian) “It depends on the learners’ proficiency. For the beginning learners, I paid more attention to their pronunciation so as to set a good foundation for their Chinese proficiency.” (Li)
Learner affect Teachers’ individual experiences	“Explicit correction is my least favored *CF*. This type of *CF* is not good protect learners’ enthusiasm, as teacher tell them that this is wrong, and that is not correct, they may lose confidence in speaking Chinese.” (Hu) “I would not like to explain grammar rules to students, given that I have not learned these rules when I was a student. Also, these rules are difficult for students to understand, so I would rather prefer to use some examples to explain students’ errors?” (Chen)
The gravity of errors Priority on the flow of students’ speech Concern for instructional pace	“I can tolerate the errors that do not cause the breakdown of communication, but when the error leads to misunderstanding, I will absolutely correct.” (Ruan) “Generally, in order to practice and improve their fluency in Chinese speaking, I would like to correct when they complete their talk.” (Zhao) “Generally I would like to correct when students finished their talk, otherwise, They might be interrupted and affected their fluency.” (Chen) “Sometimes I do not prefer immediate correction, as too much correction could disturb and slow the pace of my instruction.” (Hu)

As can be seen from Table 5, CSL teachers’ *CF* preferences relate very much to their concern for learner affect, the gravity of errors, and learner participation in the process of error correction. From the interview data, only one teacher expressed preference for explicit *CF*, while the rest (*n* = 7) expressed more favor for different techniques of implicit *CF*, regardless of error types. They argued that these *CF* types not only provided space for learners to rethink and self-identify their errors, but also propelled learners to self-repair, the process of which is conducive to learning. Another important reason was their concern about learner affect, which has been pointed out nineteen times in the interview, as all the eight teachers emphasized that their way of giving *CF* should protect students’ confidence and enthusiasm and avoid causing students’ fear, discouragement or frustration. In addition, teachers also weigh the gravity of errors when using *CF*, as six teachers were reluctant to correct all errors but focused exclusively on those that impact understanding. Also, teachers’ concern about the pace of instruction and the flow of students’ speech affects the timing for their *CF* given. As shown in the interview comments, the eight teachers expressed that their favor for delayed correction was due to their concerns about limited class time and disruption of *CF* on the flow of students’ speech. Last but not least, teachers’ individual experiences color their *CF* preferences as well. One teacher linked her dislike for metalinguistic clues to her own negative language learning experiences of not receiving grammar explanation.

#### 4.3.2. Reasons for students’ corrective feedback preferences

As for the CSL students, the results showed six reasons for their *CF* preferences (see in Table 7), Table 8 gives illuminating examples of students’ comments for each reason.

**Table 7:** Reasons for the students’ *CF* preferences.

Coding categories	*of coded S Qu	*of coded instances in S Qu
Facilitative for learning	9	13
Focusing on the entirety of speech	7	7
Affect	9	16
Helpful to identify errors	9	17
Teachers’ authoritative role	11	11
Concern for class time	5	5

*of coded S Qu refers to the number of questionnaires in which such a comment was coded; *of coded instances in S Qu refers to the number of comments that were coded, i.e., there could be several comments on a theme in one and the same questionnaire.

**Table 8:** Comments from the students for their *CF* preferences.

Coding categories	Examples
Facilitative for learning Focusing on the entirety of speech	“I thinks *CF* is very necessary. If the teacher do not correct you, you do not know what is wrong and whether you have made improvement.”(S2) “I like to be corrected explicitly, because in that way, I will know what is my error and I can remember it well.” (S7) “Correct me when I finished my talk. I will forget and lose my idea about what I am saying if someone interrupts me by correction.” (S10)
Affect Helpful to identify errors	“When the teacher said ‘please say it again’, the student might already have known something wrong with the utterances. In this sense, one may feel embarrassed to repeat the incorrect utterance again.” (S8) “The most effective one is explicit correction, but the teacher has to pay attention to their attitude, how to tell it in a friendly way. Otherwise, it will make me feel discouraged and shy. Then I do not want to speak any more.” (S14) “I prefer teachers to use explicit correction, as it can immediately tell me where and what my error is. In this way, I can remember it well and for a long time.” (S5) “For instance, if I made a mistake, the teacher used ‘clarification request’. Although I repeated my utterances many times, I may still not know whether his intention is reminding me of my errors or complementing what I said.” (S13)
Teachers’ authoritative role Concern for class time	“Teacher is professional, because we are foreigners, other classmates may make mistakes as well.” (S4) “If I said something wrong, I would like my teacher to tell me directly, because the teacher may not have enough time to prompt each student to find the error and self-repair within the limited time of the class, especially when there are many students.” (S3)

As shown in Table 7, CSL students’ *CF* preferences are ascribed to their concern about the efficacy of the given *CF* on learning, errors identification, affect, teacher role, the entirety of communication, and class time. They indicate an overall preference for explicit *CF* by arguing that this type of *CF* is most effective for their learning. For example, two interviewees attributed their preferences for explicit *CF* to its efficacy in helping them memorize the correct form and avoid committing the same error again. Moreover, eleven interviewees commented that explicit *CF* not only helped them immediately identify what and where the errors were, but also provided target reformulations or explanations. This also gave one reason for the CSL students’ less favorable attitude toward implicit *CF*, as ten of them argued that this type of *CF* confused them. They did not fully understand the teacher’s corrective intentions and failed to identify the errors, which was not conducive to learning. Another reason underlying CSL students’ *CF* preferences was born out of their concern about instructional issues, since six interviewees attributed their favor for explicit *CF* to its trait in time saving. In addition, all the student interviewees highly favored error correction by teachers because of their authoritative role and knowledge. In relation to the timing of *CF* given, seven student interviewees preferred delayed correction given that this type of *CF* allows them to focus on completing their speech without being interrupted.

Additionally, social impact is frequently mentioned by the student interviewees. For instance, five of them attributed their dislike for implicit *CF* to the fact that this type of *CF* is more likely to make them feel embarrassed by exposing them to the whole class. Also, since the teacher has to focus on the student who committed an error when using implicit *CF* they cannot engage the other students in the class which leads to boredom. Interestingly, it should be noted that in spite of CSL students’ strong preferences for explicit *CF*, three of them highlighted the need for teachers to have a care with their attitude when using these *CF* types, as explicit *CF* with an unfriendly attitude may result in students being too scared and embarrassed to speak.

In a nutshell, the interview data identified various reasons behind CSL teachers’ and students’ respective preferences for oral *CF*, which reveals more details about the discrepancies between their *CF* preferences. These results are further discussed below.

## 5. Discussion

The first research question asked about CSL teachers’ and students’ preferences for *CF* types in relation to grammatical, lexical, and phonological errors. The results added support to the existence of a discrepancy between teachers’ and students’ preferences for *CF* as reported by previous studies (Yoshida, 2008; Lee, 2013; Roothooft and Breeze, 2016). Specifically, CSL students in this study had more desire for explicit types of *CF*, which is consistent with previous studies (Roothooft and Breeze, 2016; Yang, 2016; Zhu and Wang, 2019), while teachers more preferred recasts, corroborating the findings of Lee (2013) and Yoshida (2008).

One possible explanation for CSL students’ *CF* preferences for explicit *CF* may be attributed to the characteristics of the Chinese language. As shown by Li and Zhang (2016), some distinctive features in the Chinese phonological system are difficult for students to judge and recognize, let alone make error corrections. In addition, the Chinese lexical system with its large number of synonyms is also hard for students to master them appropriately, which was well reflected in the interview data. These difficulties may have contributed to CSL students’ greater reliance on teachers when it comes to *CF* provision. This helps to explain why they appreciated explicit *CF*, since this type of *CF* entail teachers’ involvement in the error correction process. The interview data further elaborates CSL students’ preferences for explicit *CF* by arguing that this type of *CF* is effective for them to identify errors and facilitates them to memorize the correct form. Nonetheless, students expressed gratitude towards teachers using a friendly attitude when giving explicit *CF* so as to protect their positive emotional state, which is in accordance with the findings of Ha and Nguyen (2021). The message implied here is that there may be a tension between students’ pedagogical and affective concerns when it comes to how *CF* should be given. This further highlights the complexity of learners’ *CF* beliefs, as argued by Brown (2009), learners’ beliefs represent an interactive interplay of multiple variables at social, cognitive, linguistic, affective, and contextual levels.

With regard to CSL teachers’ preferences for recasts, it may lie in Chinese teachers’ entrenched beliefs about their authoritative role in classroom. Under the influence of Confucian thinking, the teacher enjoys absolute authority, and expects not to be challenged or interrupted during teaching (Li, 2003). Recasts, characterized by a teacher’s direct reformulation of learner’s error, well establish this authoritative role of Chinese teacher during class. What’s more, their unobtrusive and implicit features protect teacher from being interrupted when giving *CF*. This may explain CSL teachers’ preference for recasts instead of inducing learners to self-repair through prompts.

As for the answer to the second research question about whether there are significant differences between CSL teachers’ and students’ preferences for *CF* types, this study showed that CSL students had more preferences for clarification requests than their teachers across error types. Two explanations are suggested, one may relate to students’ proficiency level. As described above, the majority of the students (63.4%) were at least at intermediate level, while a small percentage of them (36.6%) were elementary. This majority of relatively more proficient students may explain why CSL students’ viewed clarification requests more favorably, as the better their proficiency, the greater the preference learners have for implicit *CF* (Jean and Simard, 2011; Kaivanpanah et al., 2015; Roothooft and Breeze, 2016; Yang, 2016). Another reason may be ascribed to the features of clarification requests themselves; as compared with other types of implicit *CF*. Clarification requests may be more salient due to the teacher’s clear demand for students to repeat their utterances (Lyster, 1998). It is this saliency that helps raise learners’ awareness of teacher’s corrective intentions when using a clarification request, which was also evident in the interviews, as two interviewees commented that they could become aware of something wrong in their speech immediately upon receiving a clarification request. This is perhaps another reflection of CSL students’ preferences for clarification requests, although it functions as implicit *CF*.

When it comes to the third research question regarding the reasons underlying CSL teachers’ and students’ preferences for *CF*, the results revealed diverse reasons related to their *CF* preferences, which highlights the complexity of how *CF* should be given. For instance, the interview data showed that CSL teachers’ favor for recasts relate to their concern for learner affect, as recasts, featured by unobtrusive and implicit nature, protect learners from suffering embarrassment or fear which is mostly generated by explicit *CF*. Nevertheless, the teachers also desired to use implicit *CF* such as repetition, elicitation, etc., out of their intention to encourage learners to participate in the process of error correction. This seems to suggest a tension between CSL teachers’ cultural beliefs about their dominant role during class and their intention to have learners participated. This tension projects a long-standing impact of teachers’ beliefs on changes in their teaching practices, since teachers’ beliefs “act as a gatekeeper as to whether or not to let changes happen in [the] classroom” (Mori, 2011, p. 454). In addition, given CSL students’ divergent *CF* preferences and their multiple reasons behind, it becomes highlighted that teacher should have awareness of combining different types of *CF* to better satisfy individual student’s learning needs.

In sum, the results of this study have important implications for teachers and teacher education programs. First, recasts should be provided in a salient manner so as to maximize their value. Although recasts are implicit in nature, they may become more salient for students if they are provided intensively, partially, or using a stressed intonation in order to better facilitate L2 learning (Ellis et al., 2006). Second, teachers should draw more frequently on explicit types of *CF*, but they should do so in a friendly tone to make them more acceptable for students. Third, teachers should be wary of using implicit *CF* such as clarification requests, elicitation, and repetition, since these *CF* types are more likely to confuse learners due to their ambiguity and to make learner feel embarrassed by exposing them to the whole class. With this knowledge, teachers could communicate with students in advance about their theoretical rationale and their intention when using these types of *CF*, in order for students to better understand teachers’ intentions and then react to them positively. Fourth, teachers should listen to learner voices in order to increase congruity between their *CF* practices and learners’ expectations, which would clearly make *CF* more effective for learning. As Muranoi (2000) argued, what matters for learning is not the exact type of feedback itself, but how learners approach it and use it. Finally, these results cast some light on teacher education programs by encouraging teachers to reflect on their teaching beliefs and use their insights to innovate or ameliorate their teaching practices.

## 6. Conclusion

This study investigated CSL teachers’ and students’ preferences for different types of *CF* in terms of grammatical, lexical, and phonological errors, and the reasons behind their respective *CF* preferences. The results showed CSL teachers’ overall preferences for recasts, while students preferred explicit types of *CF* across error types. In addition, teachers and students varied significantly in their preferences for some *CF* techniques. Specifically, CSL students were more favorable toward explicit types of *CF* and clarification requests, regardless of error types, while teachers appreciated recasts, especially in terms of phonological and lexical errors. Explanations for these differences may be attributed to the characteristics of the Chinese language, teachers’ culture-rooted role in classroom, student proficiency, and the features of the given *CF* type. In addition, the interview data identified various reasons behind CSL teachers’ and students’ respective preferences for *CF*. The results of this study shed light on second language teachers in terms of how to make good use of their *CF* practices to better serve teaching and learning.

As with most research, the present study also has limitations. First, the items in both questionnaires were carefully designed and the tools were piloted using sample participants, however, there still may be no guarantee that individual participants understood them clearly. Given this, there is the possibility that some responses may not have exactly reflected the perceptions of the participants. Second, the small number of participants in the interview phase may have prevented us from achieving a holistic picture of why CSL teachers and students have such different *CF* preferences. Third, participating students may vary in *CF* preferences in terms of their different cultural backgrounds and proficiencies. Further studies are warranted to examine whether there are individual differences in *CF* preferences. Fourth, it may not fully elaborate teachers’ and students’ *CF* preferences, as we have not considered the emotional load of the given *CF*, which warrants further inquiry. Finally, it remains unknown whether CSL teachers apply their *CF* preferences as reported in the data during their actual teaching, as teachers’ beliefs may not always be a reliable representation of the teaching reality in the classroom (Pajares, 1992). Given this potential mismatch, other methods such as classroom observation could be used in future research. These limitations will function as a driving force for further inquiries in *CF*-related beliefs.

## Data availability statement

The original contributions presented in the study are included in the article/Supplementary material, further inquiries can be directed to the corresponding authors.

## Author contributions

RB wrote the original manuscript. HW revised the manuscript. All authors were analyzed the data, contributed to the article, and approved the submitted version.

## Funding

This work was funded by China National Social Science Grant (NO.19BYY038).

## Conflict of interest

The authors declare that the research was conducted in the absence of any commercial or financial relationships that could be construed as a potential conflict of interest.

## Publisher’s note

All claims expressed in this article are solely those of the authors and do not necessarily represent those of their affiliated organizations, or those of the publisher, the editors and the reviewers. Any product that may be evaluated in this article, or claim that may be made by its manufacturer, is not guaranteed or endorsed by the publisher.

## Footnotes

^1^HSK is an international standardized exam used to assess non-native Chinese speakers’ abilities in using the Chinese language in their daily, academic and professional lives. HSK consists of six levels, namely from HSK1 to HSK6 in an ascending order. http://english.hanban.org/node_8002.htm

## Appendix A: Questionnaires for students and teachers

**Students’ questionnaire**

**Part 1 Background information**

1. Your nationality:

2. Your age:

A. 18–23 B. 24–29\u00B0 C. 30–35 D. 36–40 E. Over 40

3. Gender

A. Male B. Female

4. Current education:

5. Years of Chinese study:

A. 0–1 B. 2\u00B0 C. 3 D. 3–5

6. Chinese level

A. HSK1 B. HSK2 C. HSK3 D. HSK4 E. HSK5 F. HSK6

**Teachers’ questionnaire: background information**

**P art 1 Background information**

1. 您的性别:A 男 B 女

2. 您的年龄:A 18–25 B 26–30\u00B0C 31–40 D 41–50 E51-60\u00B0F 60岁以上

3. 您所学专业

4. 您的最后学历

5. 您目前从事汉语教学时间

6. 您是否有海外留学经历? A是 B 否

**Part 2**

**Please rate the level of your preferences for the following six corrective feedback each**

Grammatical error

Student: 他不有哥哥(*he does not have an older brother. Incorrect use of the verb “bu”)

Teacher:---------.

A. very like B. like C. dislike D. very dislike

1. 他…(he…)

2. 不有?(incorrect verb repetition)

3. 他没有哥哥(he has not elder brother with the correct verb “you”)

4. 这里动词是“有”(the verb here is “to have/has”)

5. 不对，应该说“他没有哥哥”(it is wrong, you should say “he has not older brother by using the correct verb”)

6. 请再说一遍?(please say it again)

Phonological error

Student: 今天很(hèn)冷(*Today is very cold. Incorrect tone of ‘很(very)’.)

Teacher:---------.

7. 恩，很冷(yes, very cold. With correct tone of 很(very).)

8. hèn?(incorrect tone repetition)

9. 不是 ‘hèn’，是 ‘hěn’,(It is not ‘hèn’, it is ‘hěn’)

10. 今天….(Today….?)

11. 注意“很”是第三声(pay attention to the tone of “很,” it should be the third tone)

12. 请再说一遍?(please say it again)

Lexical error

Student: 他去家了(*he went home, including an error in the vocabulary “去”)

Teacher:---------.

13. 去家?(incorrect word repetition)

14. 恩，他回家了(yes, he went home, with correct word)

15. 不对，应该说“他回家了” (It is not correct, you should say ‘he went home’ by correcting the wrong word ‘去’)

16. 我们说过‘家’前面的词不是去 (we mentioned before the word with ‘home’ is not ‘qu’….?)

17. 他….(he….)

18. 请再说一遍?(please say it again)

## Appendix B: Interview guidelines for teachers and students

1. Do you think *CF* is necessary? Why?

2. When do you like to receive *CF*? Why?

3. Do you think who should provide *CF*? Why?

4. To what extent do you like to be corrected? Why?

5. What types of corrective feedback do you prefer? Why?
